# Supplementary material for: Neutrophil-derived oncostatin m contributes to endothelial cell dysfunction during Treponema denticola interaction
Source: PLoS One. 2026 Oct 6;21(10):e0351640. doi: 10.1371/journal.pone.0351640 (PMC13641393; doi:10.1371/journal.pone.0351640)
Supplement: Supplementary file 1 [file pone.0351640.s001.pdf]

Fig. 2B

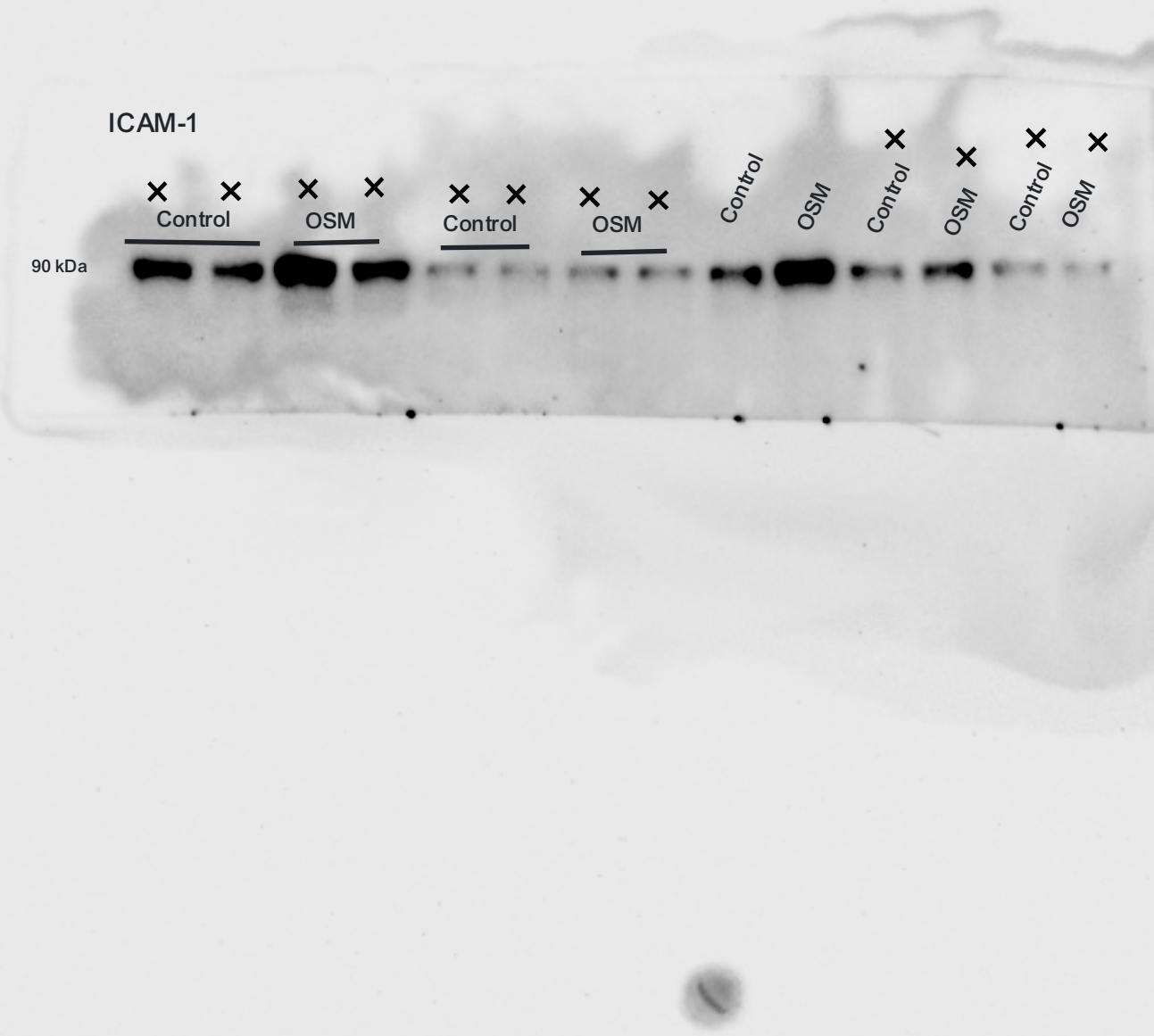
ICAM-1
90 kDa
Control
OSM
Control
OSM
Control
OSM
Control
OSM
Control
OSM

Fig. 2B

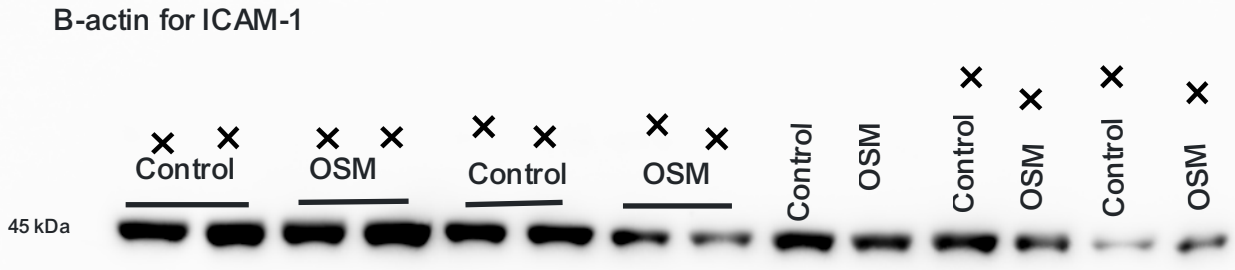
B-actin for ICAM-1
Control
OSM
Control
OSM
Control
OSM
Control
OSM
Control
OSM
45 kDa

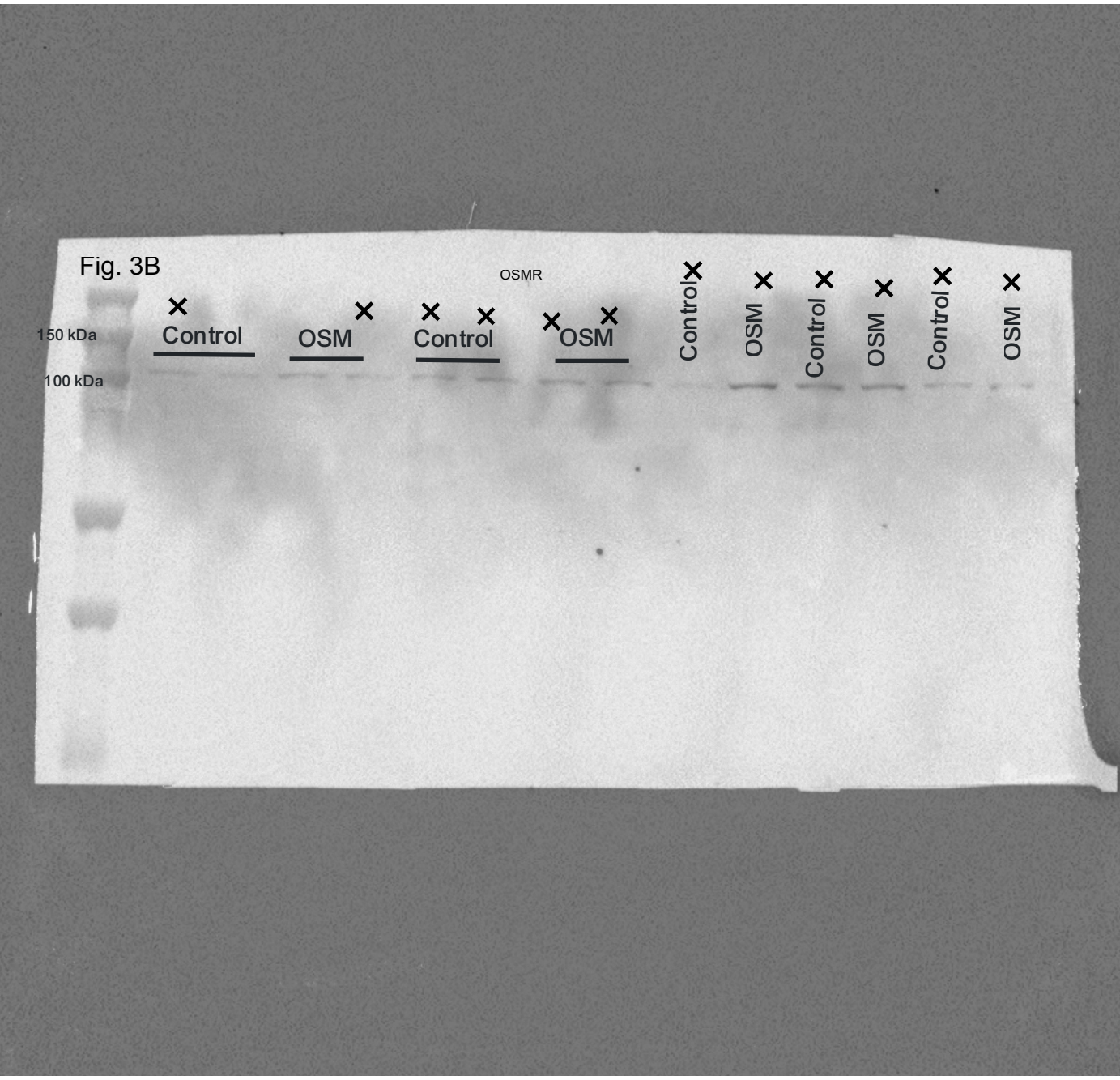
Fig. 3B
OSMR
150 kDa
100 kDa
Control
OSM
Control
OSM
Control
OSM
Control
OSM
Control
OSM

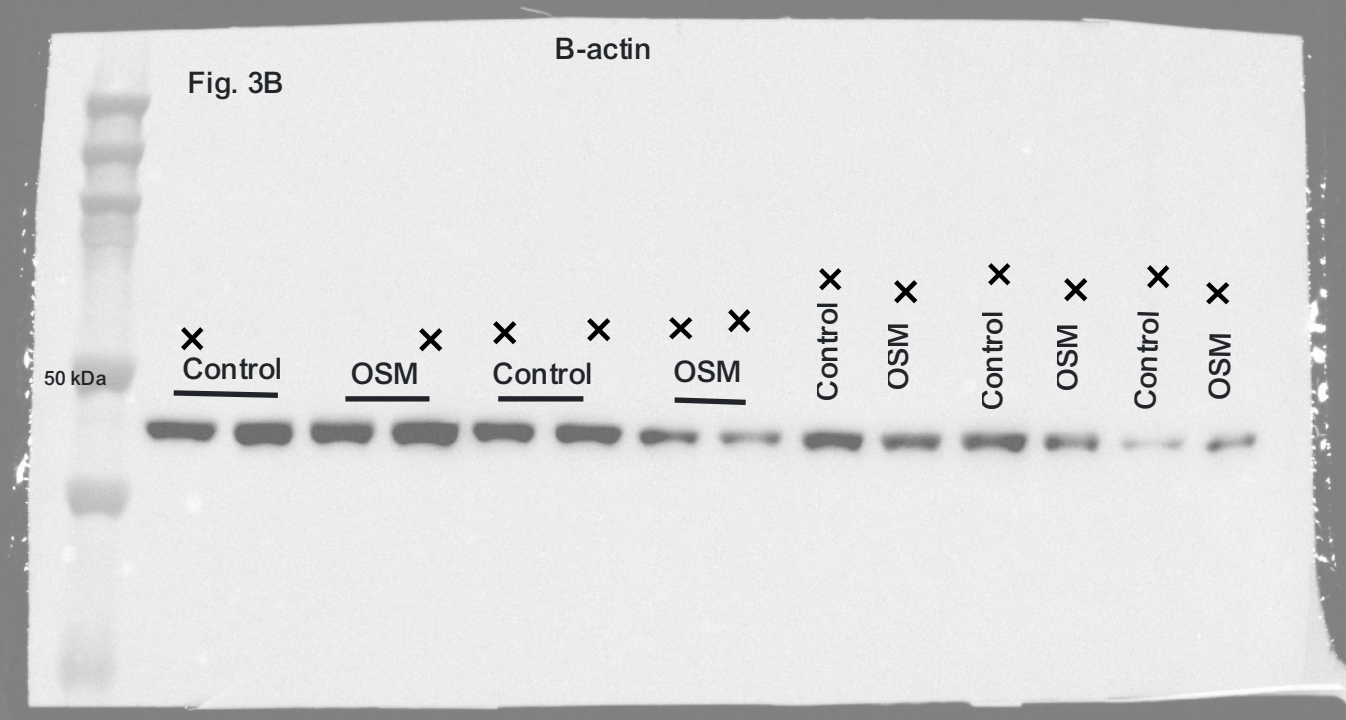
B-actin
Fig. 3B
50 kDa
Control
OSM
Control
OSM
Control
OSM
Control
OSM
Control
OSM

Fig. 3D LIFR

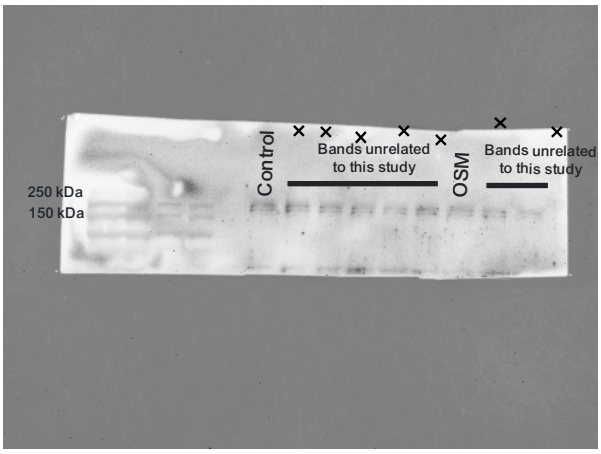

Control
Bands unrelated
to this study
OSM
Bands unrelated
to this study
250 kDa
150 kDa

Fig. 3D

B-Actin

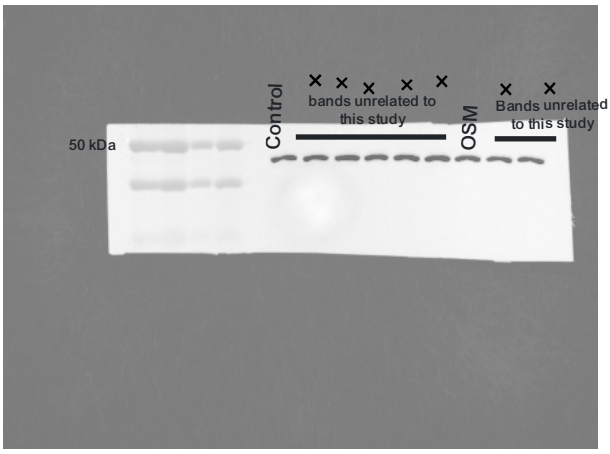

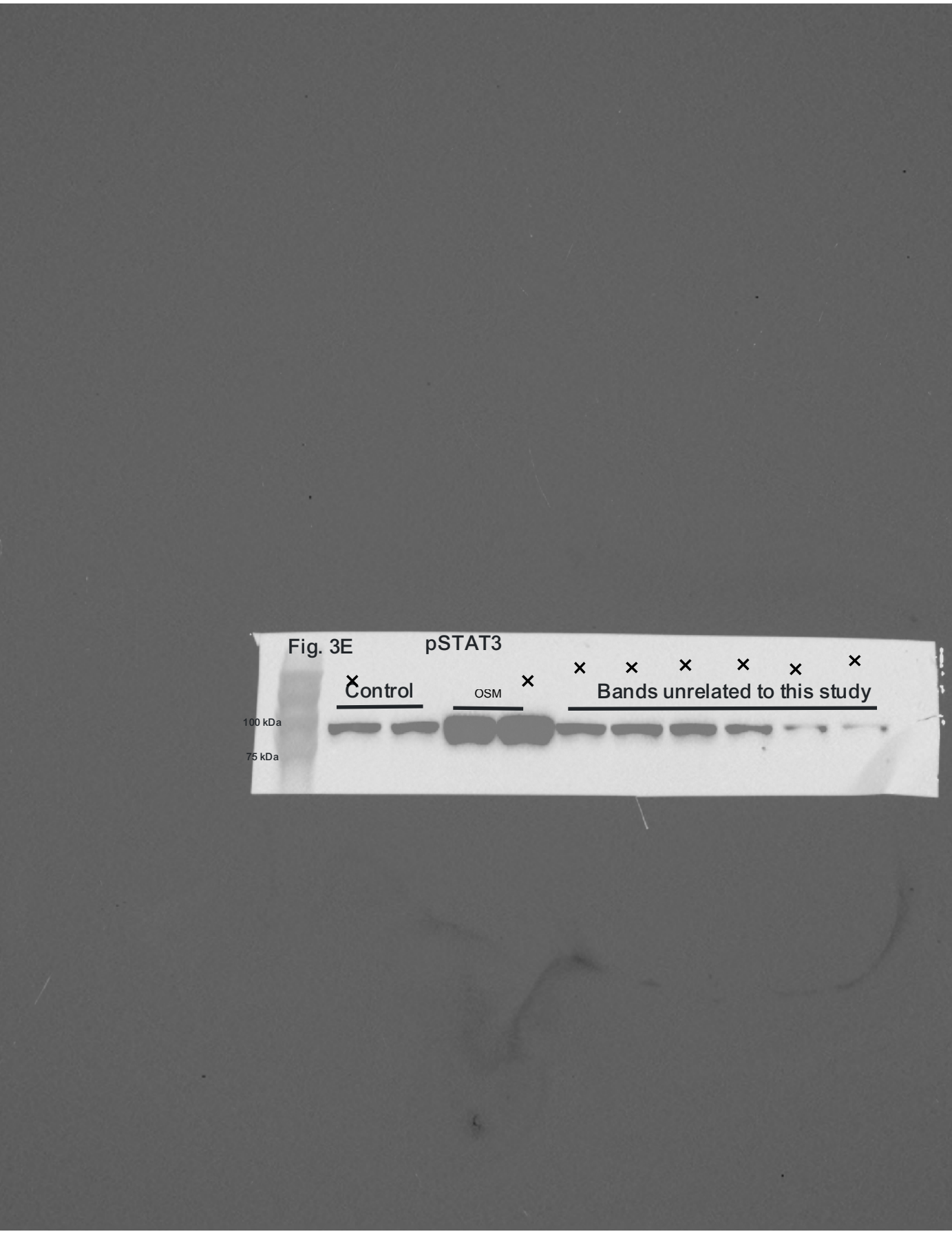

Fig. 3E
pSTAT3
Control
OSM
Bands unrelated to this study
100 kDa
75 kDa

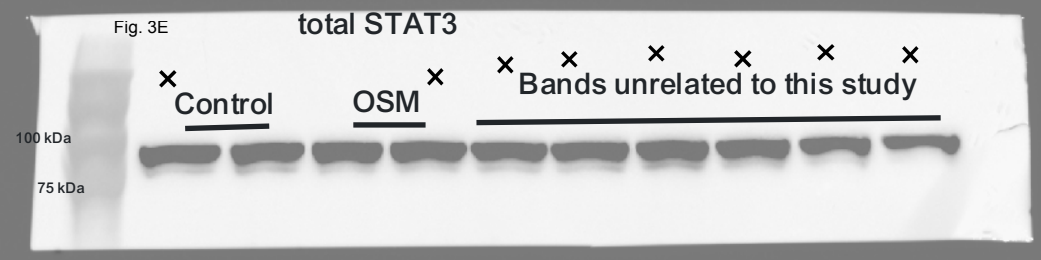
Fig. 3E
total STAT3
Control
OSM
Bands unrelated to this study
100 kDa
75 kDa

Fig. 3E

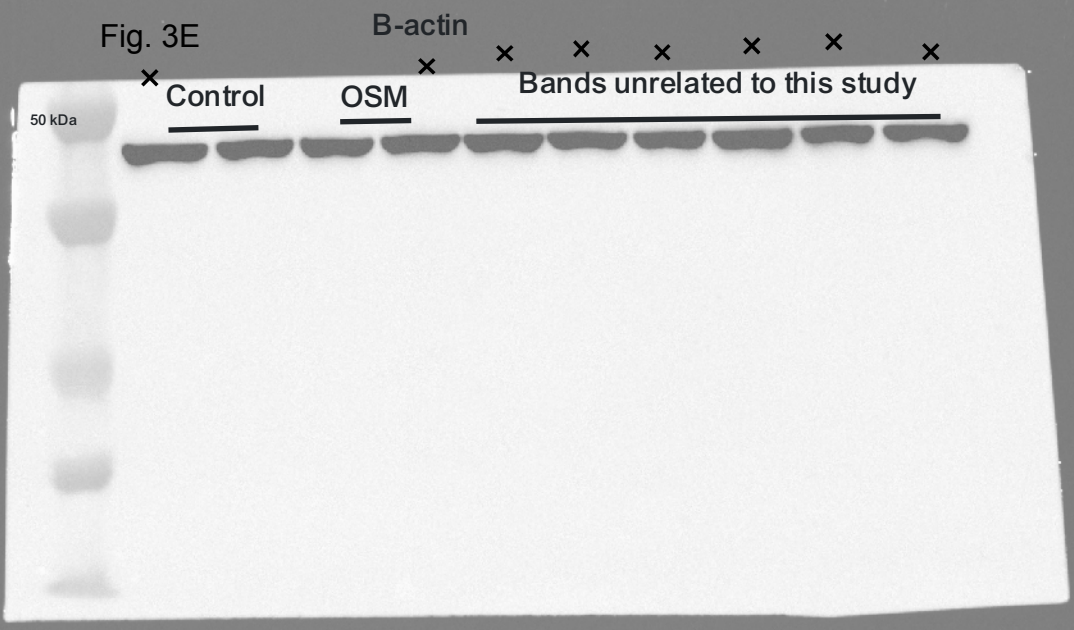

Fig. 3F

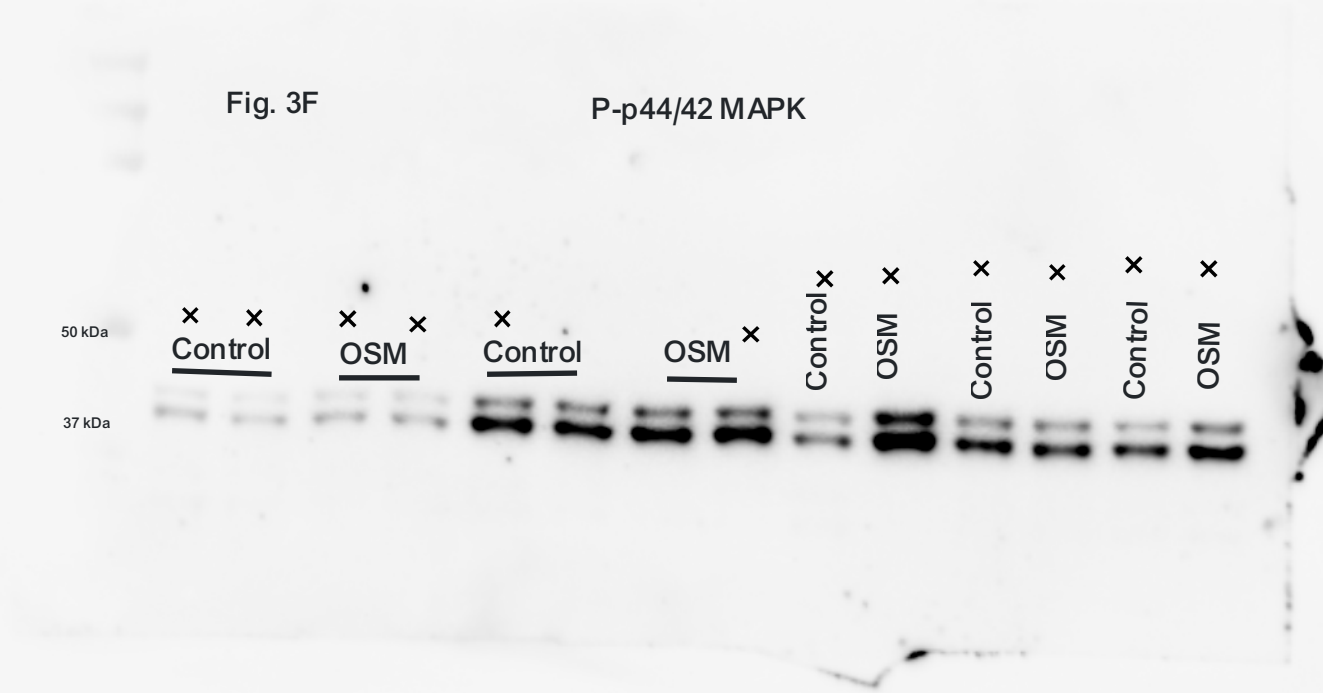
P-p44/42 MAPK
50 kDa
37 kDa
Control
OSM
Control
OSM
Control
OSM
Control
OSM
Control
OSM

Fig. 3F

p44/42 MAPK

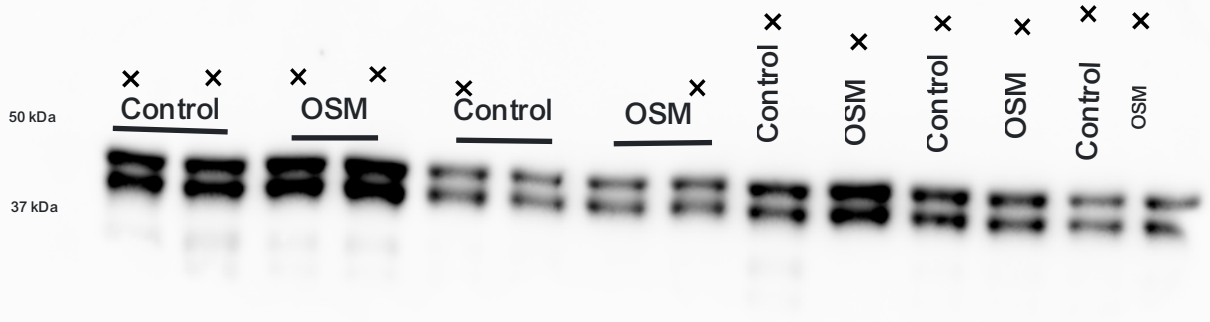

Fig. 3F

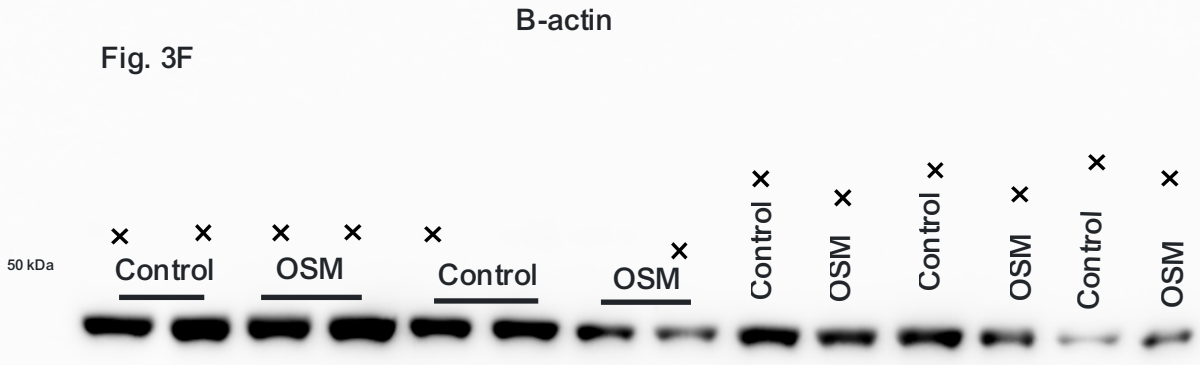
B-actin
50 kDa
Control
OSM
Control
OSM
Control
OSM
Control
OSM
Control
OSM

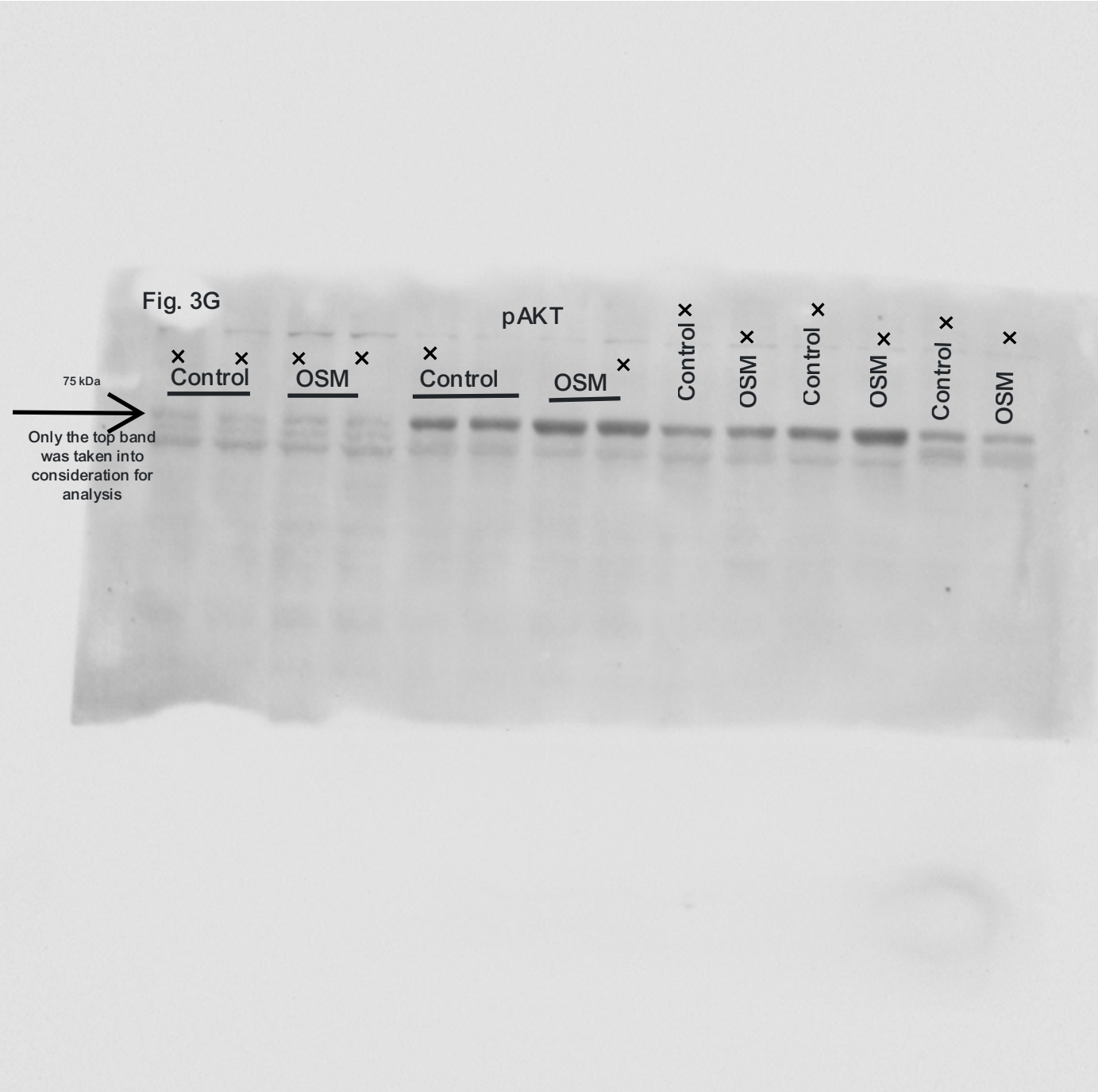

Fig. 3G
pAKT
75 kDa
Only the top band was taken into consideration for analysis
Control
OSM
Control
OSM
Control
OSM
Control
OSM
Control
OSM

Fig. 3G

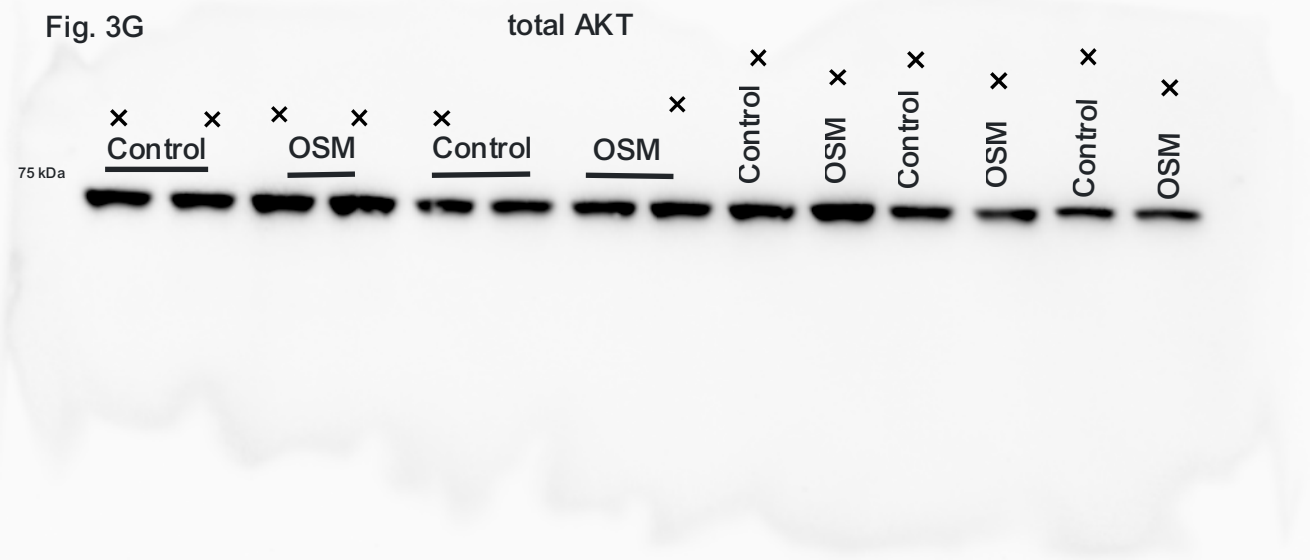
total AKT
Control
OSM
Control
OSM
Control
OSM
Control
OSM
Control
OSM
75 kDa

Fig. 3G

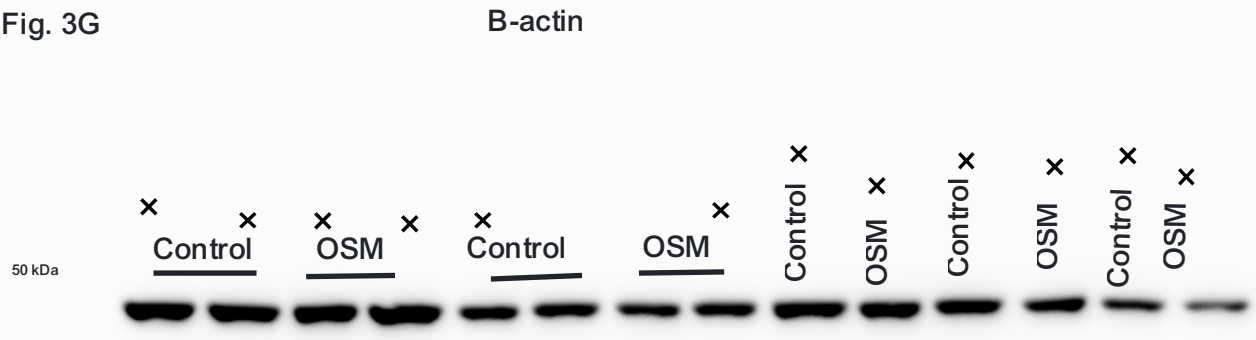

B-actin
50 kDa
Control
OSM
Control
OSM
Control
OSM
Control
OSM
Control
OSM

Fig. 4A

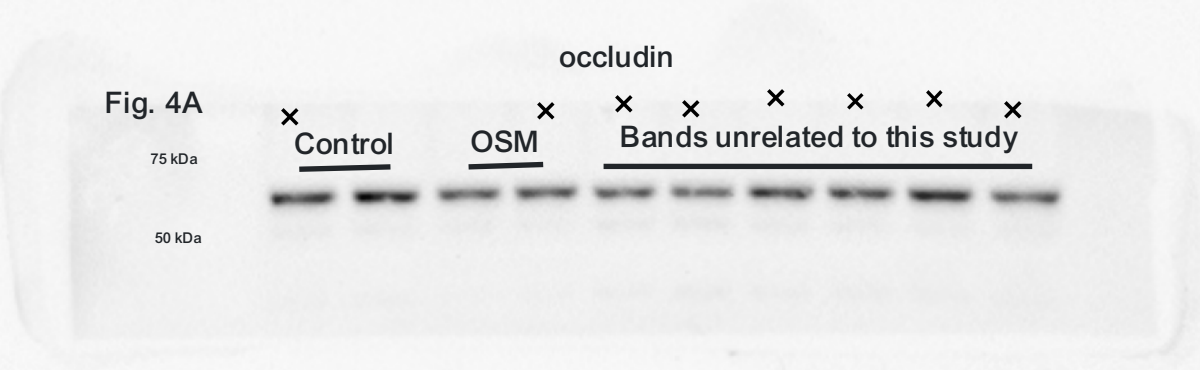

occludin
Control
OSM
Bands unrelated to this study
75 kDa
50 kDa

B-Actin

Fig. 4A

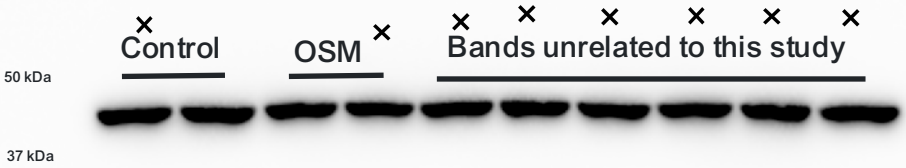

Fig. 4B

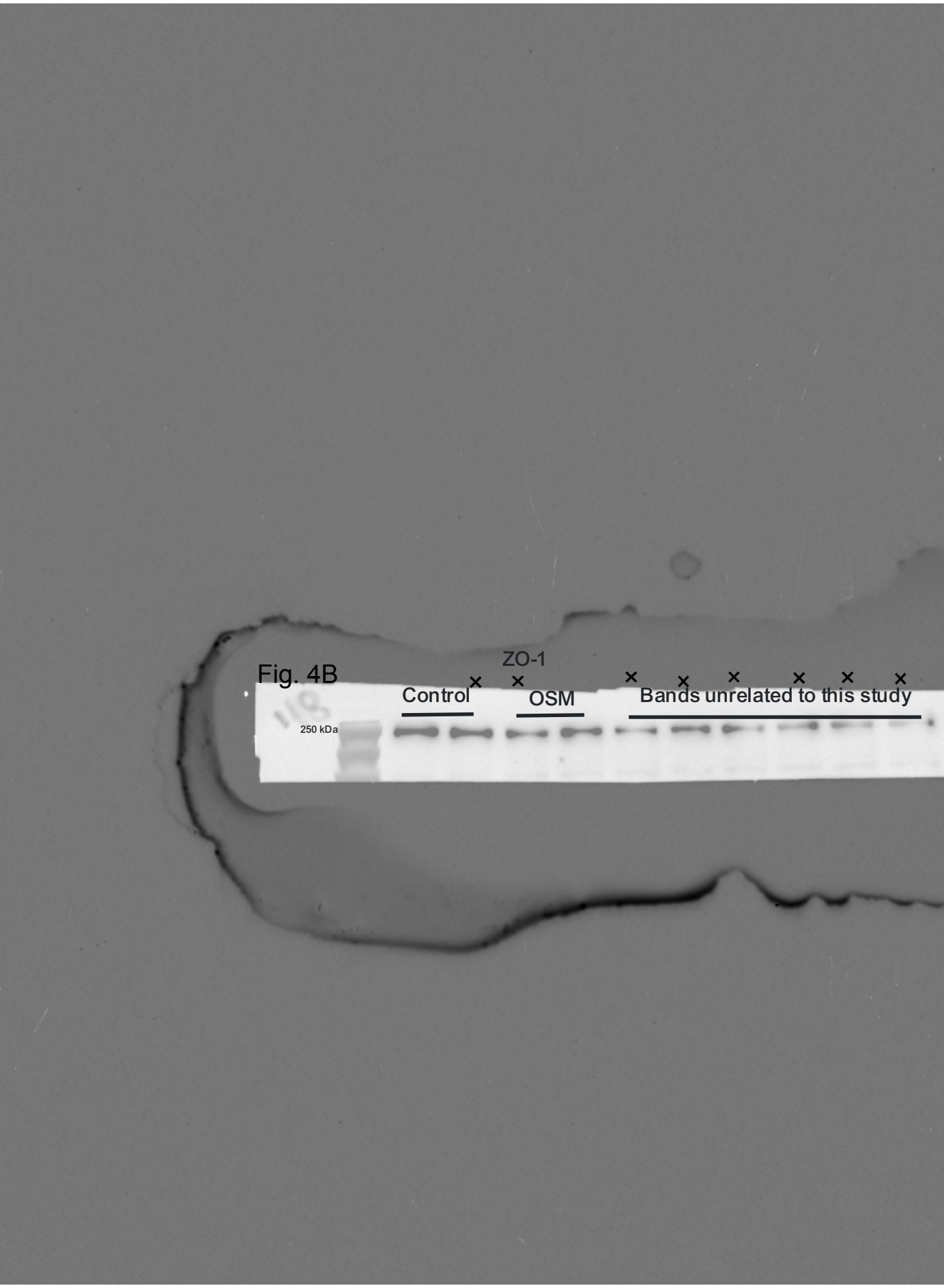

Fig. 4B

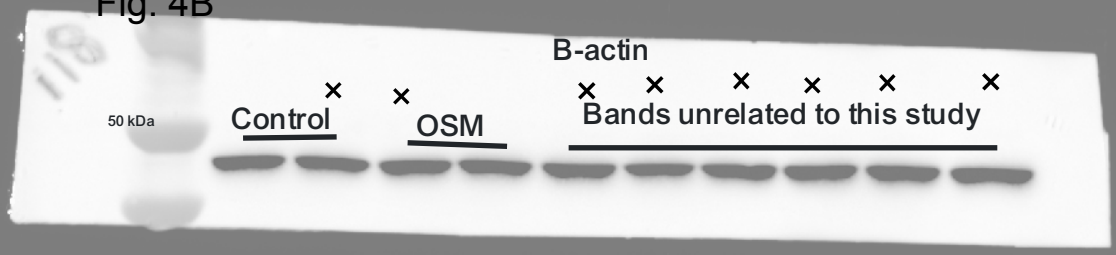

Fig. 4C

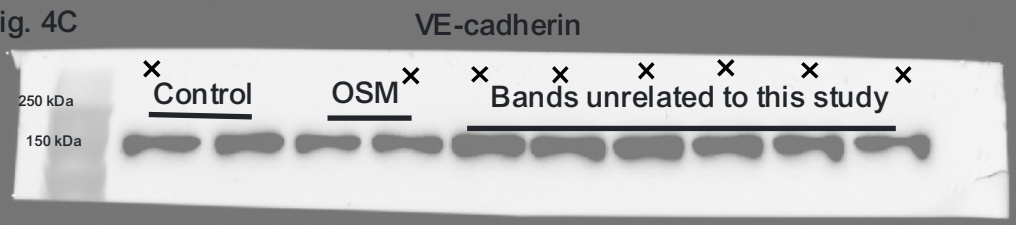

VE-cadherin
Control
OSM
Bands unrelated to this study
250 kDa
150 kDa

Fig. 4C

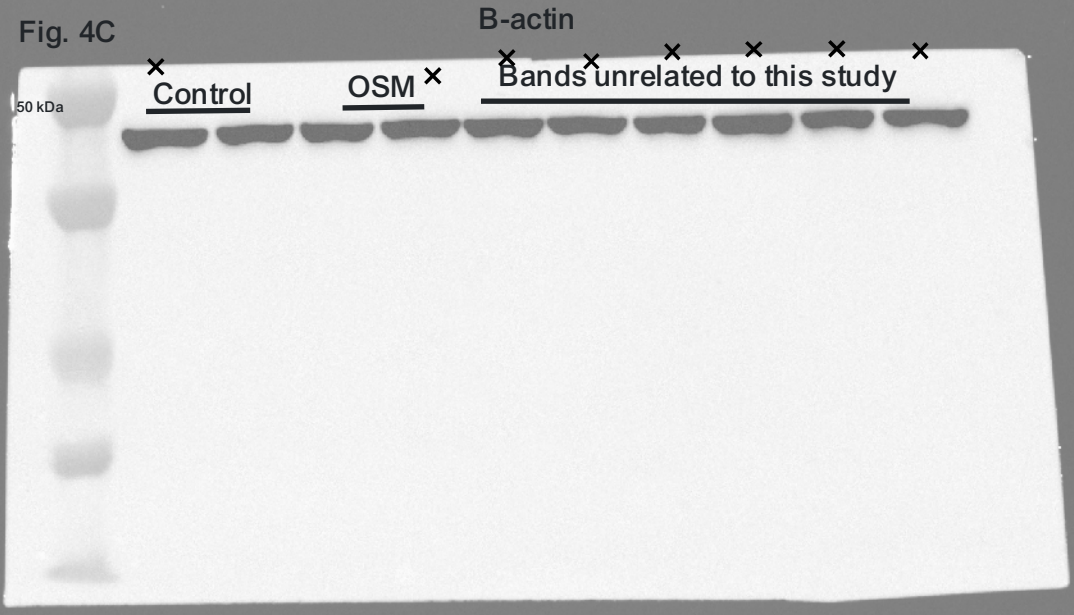

Fig. 4D

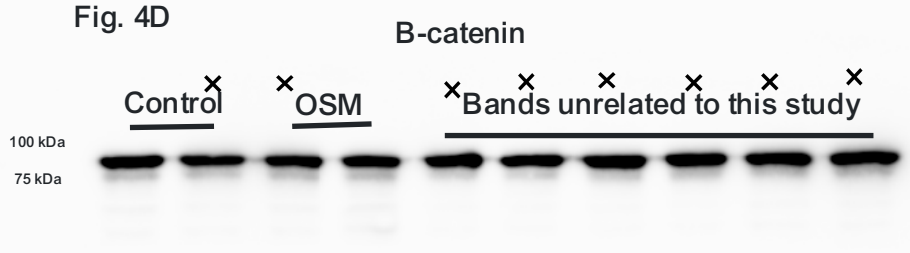
B-catenin
Control
OSM
Bands unrelated to this study
100 kDa
75 kDa

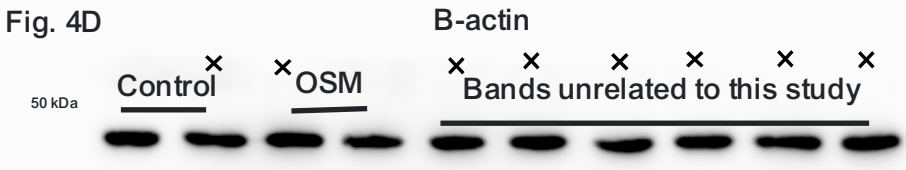
Fig. 4D
B-actin
50 kDa
Control
OSM
Bands unrelated to this study
