# Supplementary material for: Neutrophil-derived oncostatin m contributes to endothelial cell dysfunction during Treponema denticola interaction
Source: PLoS One. 2026 Oct 6;21(10):e0351640. doi: 10.1371/journal.pone.0351640 (PMC13641393; doi:10.1371/journal.pone.0351640)
Supplement: Supplementary file 2 [file pone.0351640.s004.docx]

Thank you for the thorough review of our manuscript. Below is a point by point response to all comments.

Comments:
Figure 1B: The controls for migration appear to be identical. Was this condition set to 100%? If so, that should be stated in the methods and the figure legend. Is there another way to present this data that reflects the reproducibility of the control condition? Additionally, 1000% neutrophil migration is not a real metric. Should it be a fold increase in migrated neutrophils?

This data is now presented as fold change relative to the control condition and this has been clarified in the main text and the figure legend. Details of this analysis are also updated in the methods section.

Also, please correct fmpl to fMLP in the figure legend.

This has been corrected in the legend for Figure 1.

Results regarding Figure 3: The statement that OSMR-mediated signaling to STAT3 is premature. The results showing that pSTAT3 is increased after OSM treatment are clear, but the statement that signaling through OSMR is not supported by the data. Either remove the statement that this is OSMR-mediated or provide something like siRNA knockdown or antibody antagonism of each receptor prior to OSM treatment to better demonstrate whether either receptor, or both, is mediating the STAT3 signaling.

We have edited the tone of these statements to reflect our current data (LINES 406-407, 633-634). We do agree that techniques such as siRNA or antibody antagonism would definitively address direct OSM mediated OSMR- STAT3 regulation.

Results regarding Figure 4: How do you square the Western blot data showing no increase in VE-cadherin but the IFA data showing a ~2.5 times increase in VE-cadherin detection? Both can’t be true. Also, the quantification in 4E needs to be repeated. The full n=50 will always show highly significant results, even if the biological difference is negligible. Better to report the average of different experiments with SEM.

We have reanalyzed this data in Figure 4E as suggested by this reviewer to show the mean average fluorescent intensity of the individual experiments, which is now more supportive of our western blot data in Figure 4C which shows no significant increase in total protein levels of VE-cadherin (LINES 425- 430).

The immunofluorescence imaging data still demonstrates increased change in VE-cadherin distribution at cell-cell junctions, which we propose represent alterations in the protein distribution and/or molecular changes in the molecule topology or modification events which may change the accessibility of the protein epitopes recognized by the antibody used.

Results regarding Figure 5: The title of this results section should be changed, as the statement that Td is the primary treponema species inducing OSM is not supported by the data. The results section states the K1 and MHE strains are not different from WT, but those analyses are not presented. Similarly, the text states that only Td induces hOSM, but no statistical analysis is presented to support that claim, and no difference is tested between the species. Is there qRT-PCR data to support the ELISA data? Both Td and Pg are highly proteolytic, so the ELISA values may not reflect true induction or repression. qRT-PCR data would support the protein data.

We have now changed the title of this section to “Oral Treponema species differentially induce OSM production in neutrophils” (LINE 450).

All comparative analyses between strains and species were originally performed, however, to simplify the graph images and for ease of reading, we have only showed significantly different p values in these figures. We have now included the non-significant p values in the figure legend and noted this in the Statistical Analysis section in the Methods.

We have also provided new qRT-PCR data demonstrating significant increases in *osm* gene expression following exposure to all three *Treponema* species compared to control treatment, while *P. gingivalis* does not show any significant change compared to control treatment (Figure 5C). Together with our ELISA data, this supports the conclusion that Treponema species increase OSM gene and protein expression to reflect true induction, compared to *P. gingivalis.* This has been expanded upon in the results (LINE 472-484) and discussion (LINE 718-725) sections.

Results regarding Figure 6: Again in 6B it appears the PMN condition was fixed to 100. This needs to be clearl described in the methods and the legend. Additionally, the y-axis title should reflect “relative MFI” and not as a percentage. The text states that the OSM-neutralizing antibody reduced induction permeabilization compared to the antibody control or no treatment, but those analyses are not shown. Fig. 6D-E; Again, there are descriptions without appropriate analyses shown.

All comparative analyses between treatment groups were originally performed, however, to simplify the graph images and for ease of reading, we have only showed significantly different p values in these figures. We have now updated the p values shown and included the non-significant p values in the figure legend and noted this in the Statistical Analysis section in the Methods.

Methods:
qRT-PCR methods do not mention whether no RT controls were used to assess gDNA contamination. Was this done?

Water negative controls were routinely performed within each assay, but not no RT controls in every experiment. RNA was isolated using high-quality commercial kits and primers were designed at exon-exon boundaries to minimize amplification from genomic DNA.

When submitting your revision, we need you to address these additional requirements.

1. Please ensure that your manuscript meets PLOS ONE's style requirements, including those for file naming. The PLOS ONE style templates can be found at

<https://journals.plos.org/plosone/s/file?id=wjVg/PLOSOne_formatting_sample_main_body.pdf> and 
<https://journals.plos.org/plosone/s/file?id=ba62/PLOSOne_formatting_sample_title_authors_affiliations.pdf>.

Our manuscript is now in line with journal requirements to the best of our knowledge.

2. The ethical approval number(s) listed in the manuscript and/or submission metadata does not match the approval number on the ethical approval document you provided. Please ensure that all approval numbers are correct.

The actual IRB protocol number is 030-529353 and the current approval document submitted lists the current approved modification to the protocol which is MODCR00008988. Please advise which number should be used in the manuscript?

3. Thank you for stating in your Funding Statement: 
“This work was supported by NIH/NIDCR grants R01DE027073 (MBV), F31DE033627 (DML), NIH/NIA AG083727 (MBV), NIH/NIGMS grants R25GM095459 (DML), T32GM144920 (DML) and NIH/NCATS grant UL1TR001412 (DML). The funders had no role in study design, data collection and analysis, decision to publish, or preparation of the manuscript. “Please provide an amended statement that declares *all* the funding or sources of support (whether external or internal to your organization) received during this study, as detailed online in our guide for authors at <http://journals.plos.org/plosone/s/submit-now>.  Please also include the statement “There was no additional external funding received for this study.” in your updated Funding Statement.  Please include your amended Funding Statement within your cover letter. We will change the online submission form on your behalf.

Updated Funding Statement included in cover letter.

4.  We note that your Data Availability Statement is currently as follows: [All relevant data are within the manuscript and its Supporting Information files. ]

The minimal data set of raw data is included as Supporting Information Figure 2.

5. We note that you have included the phrase “data not shown” in your manuscript. Unfortunately, this does not meet our data sharing requirements. PLOS does not permit references to inaccessible data. We require that authors provide all relevant data within the paper, Supporting Information files, or in an acceptable, public repository. Please add a citation to support this phrase or upload the data that corresponds with these findings to a stable repository (such as Figshare or Dryad) and provide and URLs, DOIs, or accession numbers that may be used to access these data. Or, if the data are not a core part of the research being presented in your study, we ask that you remove the phrase that refers to these data.

“Data not shown” has been removed and this data has now been included and expanded upon in Figure 5C.

6. PLOS ONE now requires that authors provide the original uncropped and unadjusted images underlying all blot or gel results reported in a submission’s figures or Supporting Information files. This policy and the journal’s other requirements for blot/gel reporting and figure preparation are described in detail at <https://journals.plos.org/plosone/s/figures#loc-blot-and-gel-reporting-requirements> and <https://journals.plos.org/plosone/s/figures#loc-preparing-figures-from-image-files>. When you submit your revised manuscript, please ensure that your figures adhere fully to these guidelines and provide the original underlying images for all blot or gel data reported in your submission. See the following link for instructions on providing the original image data: <https://journals.plos.org/plosone/s/figures#loc-original-images-for-blots-and-gels>.

In your cover letter, please note whether your blot/gel image data are in Supporting Information or posted at a public data repository, provide the repository URL if relevant, and provide specific details as to which raw blot/gel images, if any, are not available. Email us at [plosone@plos.org](mailto:plosone@plos.org) if you have any questions.

Original blot image data are now included as Supporting Information Figure 1 and this is noted in the cover letter.
