# Supplementary material for: Neutrophil-derived oncostatin m contributes to endothelial cell dysfunction during Treponema denticola interaction
Source: PLoS One. 2026 Oct 6;21(10):e0351640. doi: 10.1371/journal.pone.0351640 (PMC13641393; doi:10.1371/journal.pone.0351640)
Supplement: Peer review history

# Peer review: Decision Letter 0

**DOI:** 10.1371/journal.pone.0351640.r001

**Authors:** Catherine Brissette

8 Jul 2026

Dear Dr. Visser,

Thank you for submitting your manuscript to PLOS ONE. After careful consideration, we feel that it has merit but does not fully meet PLOS ONE’s publication criteria as it currently stands. Therefore, we invite you to submit a revised version of the manuscript that addresses the points raised during the review process.

==============================

Despite my best efforts, I was unable to recruit a second reviewer. However, I have have looked over the manuscript carefully and am in concurrence with Reviewer #1 regarding needed clarifications about the interpretation of the data, particularly with Fig 3 and Fig 5.

==============================

Please submit your revised manuscript by Aug 22 2026 11:59PM. If you will need more time than this to complete your revisions, please reply to this message or contact the journal office at plosone@plos.org. When you're ready to submit your revision, log on to https://www.editorialmanager.com/pone/ and select the 'Submissions Needing Revision' folder to locate your manuscript file.

- A letter that responds to each point raised by the academic editor and reviewer(s). You should upload this letter as a separate file labeled 'Response to Reviewers'.
- A marked-up copy of your manuscript that highlights changes made to the original version. You should upload this as a separate file labeled 'Revised Manuscript with Track Changes'.
- An unmarked version of your revised paper without tracked changes. You should upload this as a separate file labeled 'Manuscript'.

If you would like to make changes to your financial disclosure, please include your updated statement in your cover letter. Guidelines for resubmitting your figure files are available below the reviewer comments at the end of this letter.

If applicable, we recommend that you deposit your laboratory protocols in protocols.io to enhance the reproducibility of your results. Protocols.io assigns your protocol its own identifier (DOI) so that it can be cited independently in the future. For instructions see: https://journals.plos.org/plosone/s/submission-guidelines#loc-laboratory-protocols. Additionally, PLOS ONE offers an option for publishing peer-reviewed Lab Protocol articles, which describe protocols hosted on protocols.io. Read more information on sharing protocols at https://plos.org/protocols?utm_medium=editorial-email&utm_source=authorletters&utm_campaign=protocols.

As the corresponding author, your ORCID iD is verified in the submission system and will appear in the published article. PLOS supports the use of ORCID, and we encourage all coauthors to register for an ORCID iD and use it as well. Please encourage your coauthors to verify their ORCID iD within the submission system before final acceptance, as unverified ORCID iDs will not appear in the published article. *Only*  the individual author can complete the verification step; PLOS staff *cannot*  verify ORCID iDs on behalf of authors.

We look forward to receiving your revised manuscript.

Kind regards,

Catherine A. Brissette, Ph.D.

Academic Editor

PLOS One

Journal Requirements:

When submitting your revision, we need you to address these additional requirements.

1. Please ensure that your manuscript meets PLOS ONE's style requirements, including those for file naming. The PLOS ONE style templates can be found at

https://journals.plos.org/plosone/s/file?id=wjVg/PLOSOne_formatting_sample_main_body.pdf and

https://journals.plos.org/plosone/s/file?id=ba62/PLOSOne_formatting_sample_title_authors_affiliations.pdf.

2. The ethical approval number(s) listed in the manuscript and/or submission metadata does not match the approval number on the ethical approval document you provided. Please ensure that all approval numbers are correct.

3. Thank you for stating in your Funding Statement:

“This work was supported by NIH/NIDCR grants R01DE027073 (MBV), F31DE033627 (DML), NIH/NIA AG083727 (MBV), NIH/NIGMS grants R25GM095459 (DML), T32GM144920 (DML) and NIH/NCATS grant UL1TR001412 (DML).

The funders had no role in study design, data collection and analysis, decision to publish, or preparation of the manuscript. “

Please provide an amended statement that declares *all* the funding or sources of support (whether external or internal to your organization) received during this study, as detailed online in our guide for authors at http://journals.plos.org/plosone/s/submit-now.  Please also include the statement “There was no additional external funding received for this study.” in your updated Funding Statement.

Please include your amended Funding Statement within your cover letter. We will change the online submission form on your behalf.

4.  We note that your Data Availability Statement is currently as follows: [All relevant data are within the manuscript and its Supporting Information files. ]

Please confirm at this time whether or not your submission contains all raw data required to replicate the results of your study. Authors must share the “minimal data set” for their submission. PLOS defines the minimal data set to consist of the data required to replicate all study findings reported in the article, as well as related metadata and methods (https://journals.plos.org/plosone/s/data-availability#loc-minimal-data-set-definition).

For example, authors should submit the following data:

- The values behind the means, standard deviations and other measures reported;

- The values used to build graphs;

- The points extracted from images for analysis.

Authors do not need to submit their entire data set if only a portion of the data was used in the reported study.

If your submission does not contain these data, please either upload them as Supporting Information files or deposit them to a stable, public repository and provide us with the relevant URLs, DOIs, or accession numbers. For a list of recommended repositories, please see https://journals.plos.org/plosone/s/recommended-repositories.

If there are ethical or legal restrictions on sharing a de-identified data set, please explain them in detail (e.g., data contain potentially sensitive information, data are owned by a third-party organization, etc.) and who has imposed them (e.g., an ethics committee). Please also provide contact information for a data access committee, ethics committee, or other institutional body to which data requests may be sent. If data are owned by a third party, please indicate how others may request data access.

5. We note that you have included the phrase “data not shown” in your manuscript. Unfortunately, this does not meet our data sharing requirements. PLOS does not permit references to inaccessible data. We require that authors provide all relevant data within the paper, Supporting Information files, or in an acceptable, public repository. Please add a citation to support this phrase or upload the data that corresponds with these findings to a stable repository (such as Figshare or Dryad) and provide and URLs, DOIs, or accession numbers that may be used to access these data. Or, if the data are not a core part of the research being presented in your study, we ask that you remove the phrase that refers to these data.

6. PLOS ONE now requires that authors provide the original uncropped and unadjusted images underlying all blot or gel results reported in a submission’s figures or Supporting Information files. This policy and the journal’s other requirements for blot/gel reporting and figure preparation are described in detail at https://journals.plos.org/plosone/s/figures#loc-blot-and-gel-reporting-requirements and https://journals.plos.org/plosone/s/figures#loc-preparing-figures-from-image-files. When you submit your revised manuscript, please ensure that your figures adhere fully to these guidelines and provide the original underlying images for all blot or gel data reported in your submission. See the following link for instructions on providing the original image data: https://journals.plos.org/plosone/s/figures#loc-original-images-for-blots-and-gels.

In your cover letter, please note whether your blot/gel image data are in Supporting Information or posted at a public data repository, provide the repository URL if relevant, and provide specific details as to which raw blot/gel images, if any, are not available. Email us at plosone@plos.org if you have any questions.

7. If the reviewer comments include a recommendation to cite specific previously published works, please review and evaluate these publications to determine whether they are relevant and should be cited. There is no requirement to cite these works unless the editor has indicated otherwise.

8. Please review your reference list to ensure that it is complete and correct. If you have cited papers that have been retracted, please include the rationale for doing so in the manuscript text, or remove these references and replace them with relevant current references. Any changes to the reference list should be mentioned in the rebuttal letter that accompanies your revised manuscript. If you need to cite a retracted article, indicate the article’s retracted status in the References list and also include a citation and full reference for the retraction notice.

Additional Editor Comments (if provided):

Despite my best efforts, I was unable to recruit a second reviewer. However, I have have looked over the manuscript carefully and am in concurrence with Reviewer #1 regarding needed clarifications about the interpretation of the data.

[Note: HTML markup is below. Please do not edit.]

Reviewers' comments:

Reviewer's Responses to Questions

**Comments to the Author**

1. Is the manuscript technically sound, and do the data support the conclusions?

Reviewer #1: Partly

**********

2. Has the statistical analysis been performed appropriately and rigorously?

Reviewer #1: No

**********

3. Have the authors made all data underlying the findings in their manuscript fully available?

The PLOS Data policy

Reviewer #1: Yes

**********

4. Is the manuscript presented in an intelligible fashion and written in standard English?

Reviewer #1: Yes

**********

Reviewer #1: This is an interesting study of oral treponeme-induced oncostatin M production by neutrophils. It builds on several previous studies. The manuscript is clearly written and well presented. Some changes are needed to better support the conclusions before the manuscript can be accepted.

Comments:

Figure 1B: The controls for migration appear to be identical. Was this condition set to 100%? If so, that should be stated in the methods and the figure legend. Is there another way to present this data that reflects the reproducibility of the control condition? Additionally, 1000% neutrophil migration is not a real metric. Should it be a fold increase in migrated neutrophils?

Also, please correct fmpl to fMLP in the figure legend.

Results regarding Figure 3: The statement that OSMR-mediated signaling to STAT3 is premature. The results showing that pSTAT3 is increased after OSM treatment are clear, but the statement that signaling through OSMR is not supported by the data. Either remove the statement that this is OSMR-mediated or provide something like siRNA knockdown or antibody antagonism of each receptor prior to OSM treatment to better demonstrate whether either receptor, or both, is mediating the STAT3 signaling.

Results regarding Figure 4: How do you square the Western blot data showing no increase in VE-cadherin but the IFA data showing a ~2.5 times increase in VE-cadherin detection? Both can’t be true. Also, the quantification in 4E needs to be repeated. The full n=50 will always show highly significant results, even if the biological difference is negligible. Better to report the average of different experiments with SEM.

Results regarding Figure 5: The title of this results section should be changed, as the statement that Td is the primary treponema species inducing OSM is not supported by the data. The results section states the K1 and MHE strains are not different from WT, but those analyses are not presented. Similarly, the text states that only Td induces hOSM, but no statistical analysis is presented to support that claim, and no difference is tested between the species. Is there qRT-PCR data to support the ELISA data? Both Td and Pg are highly proteolytic, so the ELISA values may not reflect true induction or repression. qRT-PCR data would support the protein data.

Results regarding Figure 6: Again in 6B it appears the PMN condition was fixed to 100. This needs to be clearl described in the methods and the legend. Additionally, the y-axis title should reflect “relative MFI” and not as a percentage. The text states that the OSM-neutralizing antibody reduced induction permeabilization compared to the antibody control or no treatment, but those analyses are not shown. Fig. 6D-E; Again, there are descriptions without appropriate analyses shown.

Methods:

qRT-PCR methods do not mention whether no RT controls were used to assess gDNA contamination. Was this done?

**********

what does this mean?). If published, this will include your full peer review and any attached files.

If you choose “no”, your identity will remain anonymous but your review may still be made public.

**Do you want your identity to be public for this peer review?**  For information about this choice, including consent withdrawal, please see our Privacy Policy

Reviewer #1: **Yes:**  Daniel P. Miller

**********

[NOTE: If reviewer comments were submitted as an attachment file, they will be attached to this email and accessible via the submission site. Please log into your account, locate the manuscript record, and check for the action link "View Attachments". If this link does not appear, there are no attachment files.]

To ensure your figures meet our technical requirements, please review our figure guidelines: https://journals.plos.org/plosone/s/figures

You may also use PLOS’s free figure tool, NAAS, to help you prepare publication quality figures: https://journals.plos.org/plosone/s/figures#loc-tools-for-figure-preparation.

NAAS will assess whether your figures meet our technical requirements by comparing each figure against our figure specifications.

# Author comment: Author response to Decision Letter 1

**DOI:** 10.1371/journal.pone.0351640.r002

16 Sep 2026

Thank you for the thorough review of our manuscript. Below is a point by point response to all comments.

Comments:

Figure 1B: The controls for migration appear to be identical. Was this condition set to 100%? If so, that should be stated in the methods and the figure legend. Is there another way to present this data that reflects the reproducibility of the control condition? Additionally, 1000% neutrophil migration is not a real metric. Should it be a fold increase in migrated neutrophils?

This data is now presented as fold change relative to the control condition and this has been clarified in the main text and the figure legend. Details of this analysis are also updated in the methods section.

Also, please correct fmpl to fMLP in the figure legend.

This has been corrected in the legend for Figure 1.

Results regarding Figure 3: The statement that OSMR-mediated signaling to STAT3 is premature. The results showing that pSTAT3 is increased after OSM treatment are clear, but the statement that signaling through OSMR is not supported by the data. Either remove the statement that this is OSMR-mediated or provide something like siRNA knockdown or antibody antagonism of each receptor prior to OSM treatment to better demonstrate whether either receptor, or both, is mediating the STAT3 signaling.

We have edited the tone of these statements to reflect our current data (LINES 406-407, 633-634). We do agree that techniques such as siRNA or antibody antagonism would definitively address direct OSM mediated OSMR- STAT3 regulation.

Results regarding Figure 4: How do you square the Western blot data showing no increase in VE-cadherin but the IFA data showing a ~2.5 times increase in VE-cadherin detection? Both can’t be true. Also, the quantification in 4E needs to be repeated. The full n=50 will always show highly significant results, even if the biological difference is negligible. Better to report the average of different experiments with SEM.

We have reanalyzed this data in Figure 4E as suggested by this reviewer to show the mean average fluorescent intensity of the individual experiments, which is now more supportive of our western blot data in Figure 4C which shows no significant increase in total protein levels of VE-cadherin (LINES 425- 430).

The immunofluorescence imaging data still demonstrates increased change in VE-cadherin distribution at cell-cell junctions, which we propose represent alterations in the protein distribution and/or molecular changes in the molecule topology or modification events which may change the accessibility of the protein epitopes recognized by the antibody used.

Results regarding Figure 5: The title of this results section should be changed, as the statement that Td is the primary treponema species inducing OSM is not supported by the data. The results section states the K1 and MHE strains are not different from WT, but those analyses are not presented. Similarly, the text states that only Td induces hOSM, but no statistical analysis is presented to support that claim, and no difference is tested between the species. Is there qRT-PCR data to support the ELISA data? Both Td and Pg are highly proteolytic, so the ELISA values may not reflect true induction or repression. qRT-PCR data would support the protein data.

We have now changed the title of this section to “Oral Treponema species differentially induce OSM production in neutrophils” (LINE 450).

All comparative analyses between strains and species were originally performed, however, to simplify the graph images and for ease of reading, we have only showed significantly different p values in these figures. We have now included the non-significant p values in the figure legend and noted this in the Statistical Analysis section in the Methods.

We have also provided new qRT-PCR data demonstrating significant increases in osm gene expression following exposure to all three Treponema species compared to control treatment, while P. gingivalis does not show any significant change compared to control treatment (Figure 5C). Together with our ELISA data, this supports the conclusion that Treponema species increase OSM gene and protein expression to reflect true induction, compared to P. gingivalis. This has been expanded upon in the results (LINE 472-484) and discussion (LINE 718-725) sections.

Results regarding Figure 6: Again in 6B it appears the PMN condition was fixed to 100. This needs to be clearl described in the methods and the legend. Additionally, the y-axis title should reflect “relative MFI” and not as a percentage. The text states that the OSM-neutralizing antibody reduced induction permeabilization compared to the antibody control or no treatment, but those analyses are not shown. Fig. 6D-E; Again, there are descriptions without appropriate analyses shown.

All comparative analyses between treatment groups were originally performed, however, to simplify the graph images and for ease of reading, we have only showed significantly different p values in these figures. We have now updated the p values shown and included the non-significant p values in the figure legend and noted this in the Statistical Analysis section in the Methods.

Methods:

qRT-PCR methods do not mention whether no RT controls were used to assess gDNA contamination. Was this done?

Water negative controls were routinely performed within each assay, but not no RT controls in every experiment. RNA was isolated using high-quality commercial kits and primers were designed at exon-exon boundaries to minimize amplification from genomic DNA.

When submitting your revision, we need you to address these additional requirements.

1. Please ensure that your manuscript meets PLOS ONE's style requirements, including those for file naming. The PLOS ONE style templates can be found at

https://journals.plos.org/plosone/s/file?id=wjVg/PLOSOne_formatting_sample_main_body.pdf and

https://journals.plos.org/plosone/s/file?id=ba62/PLOSOne_formatting_sample_title_authors_affiliations.pdf.

Our manuscript is now in line with journal requirements to the best of our knowledge.

2. The ethical approval number(s) listed in the manuscript and/or submission metadata does not match the approval number on the ethical approval document you provided. Please ensure that all approval numbers are correct.

The actual IRB protocol number is 030-529353 and the current approval document submitted lists the current approved modification to the protocol which is MODCR00008988. Please advise which number should be used in the manuscript?

3. Thank you for stating in your Funding Statement:

“This work was supported by NIH/NIDCR grants R01DE027073 (MBV), F31DE033627 (DML), NIH/NIA AG083727 (MBV), NIH/NIGMS grants R25GM095459 (DML), T32GM144920 (DML) and NIH/NCATS grant UL1TR001412 (DML). The funders had no role in study design, data collection and analysis, decision to publish, or preparation of the manuscript. “Please provide an amended statement that declares *all* the funding or sources of support (whether external or internal to your organization) received during this study, as detailed online in our guide for authors at http://journals.plos.org/plosone/s/submit-now. Please also include the statement “There was no additional external funding received for this study.” in your updated Funding Statement. Please include your amended Funding Statement within your cover letter. We will change the online submission form on your behalf.

Updated Funding Statement included in cover letter.

4. We note that your Data Availability Statement is currently as follows: [All relevant data are within the manuscript and its Supporting Information files. ]

The minimal data set of raw data is included as Supporting Information Figure 2.

5. We note that you have included the phrase “data not shown” in your manuscript. Unfortunately, this does not meet our data sharing requirements. PLOS does not permit references to inaccessible data. We require that authors provide all relevant data within the paper, Supporting Information files, or in an acceptable, public repository. Please add a citation to support this phrase or upload the data that corresponds with these findings to a stable repository (such as Figshare or Dryad) and provide and URLs, DOIs, or accession numbers that may be used to access these data. Or, if the data are not a core part of the research being presented in your study, we ask that you remove the phrase that refers to these data.

“Data not shown” has been removed and this data has now been included and expanded upon in Figure 5C.

6. PLOS ONE now requires that authors provide the original uncropped and unadjusted images underlying all blot or gel results reported in a submission’s figures or Supporting Information files. This policy and the journal’s other requirements for blot/gel reporting and figure preparation are described in detail at https://journals.plos.org/plosone/s/figures#loc-blot-and-gel-reporting-requirements and https://journals.plos.org/plosone/s/figures#loc-preparing-figures-from-image-files. When you submit your revised manuscript, please ensure that your figures adhere fully to these guidelines and provide the original underlying images for all blot or gel data reported in your submission. See the following link for instructions on providing the original image data: https://journals.plos.org/plosone/s/figures#loc-original-images-for-blots-and-gels.

In your cover letter, please note whether your blot/gel image data are in Supporting Information or posted at a public data repository, provide the repository URL if relevant, and provide specific details as to which raw blot/gel images, if any, are not available. Email us at plosone@plos.org if you have any questions.

Original blot image data are now included as Supporting Information Figure 1 and this is noted in the cover letter.

Attachment Submitted filename: Response to Reviewers.docx

# Editor report: Decision Letter 1

**DOI:** 10.1371/journal.pone.0351640.r003

**Authors:** Catherine Brissette

21 Sep 2026

Neutrophil-Derived Oncostatin M Contributes to Endothelial Cell Dysfunction During Treponema denticola interaction

PONE-D-26-18925R1

Dear Dr. Visser,

We’re pleased to inform you that your manuscript has been judged scientifically suitable for publication and will be formally accepted for publication once it meets all outstanding technical requirements.

Within one week, you’ll receive an e-mail detailing the required amendments. When these have been addressed, you’ll receive a formal acceptance letter and your manuscript will be scheduled for publication.

An invoice will be generated when your article is formally accepted. Please note, if your institution has a publishing partnership with PLOS and your article meets the relevant criteria, all or part of your publication costs will be covered. Please make sure your user information is up-to-date by logging into Editorial Manager at Editorial Manager® and clicking the ‘Update My Information' link at the top of the page. For questions related to billing, please contact billing support.

If your institution or institutions have a press office, please notify them about your upcoming paper to help maximize its impact. If they’ll be preparing press materials, please inform our press team as soon as possible -- no later than 48 hours after receiving the formal acceptance. Your manuscript will remain under strict press embargo until 2 pm Eastern Time on the date of publication. For more information, please contact onepress@plos.org.

Kind regards,

Catherine A. Brissette, Ph.D.

Academic Editor

PLOS One

Additional Editor Comments (optional):

Reviewers' comments:

# Editor report: Acceptance letter

**DOI:** 10.1371/journal.pone.0351640.r004

**Authors:** Catherine Brissette

PONE-D-26-18925R1

PLOS One

Dear Dr. Visser,

I'm pleased to inform you that your manuscript has been deemed suitable for publication in PLOS One. Congratulations! Your manuscript is now being handed over to our production team.

At this stage, our production department will prepare your paper for publication. This includes ensuring the following:

* All references, tables, and figures are properly cited

* All relevant supporting information is included in the manuscript submission,

* There are no issues that prevent the paper from being properly typeset

You will receive further instructions from the production team, including instructions on how to review your proof when it is ready. Please keep in mind that we are working through a large volume of accepted articles, so please give us a few days to review your paper and let you know the next and final steps.

Lastly, if your institution or institutions have a press office, please let them know about your upcoming paper now to help maximize its impact. If they'll be preparing press materials, please inform our press team within the next 48 hours. Your manuscript will remain under strict press embargo until 2 pm Eastern Time on the date of publication. For more information, please contact onepress@plos.org.

You will receive an invoice from PLOS for your publication fee after your manuscript has reached the completed accept phase. If you receive an email requesting payment before acceptance or for any other service, this may be a phishing scheme. Learn how to identify phishing emails and protect your accounts at https://explore.plos.org/phishing.

If we can help with anything else, please email us at customercare@plos.org.

Thank you for submitting your work to PLOS One and supporting open access.

Kind regards,

PLOS One Editorial Office Staff

on behalf of

Dr. Catherine A. Brissette

Academic Editor

PLOS One
